# miR-29c-3p regulates DNMT3B and LATS1 methylation to inhibit tumor progression in hepatocellular carcinoma

**DOI:** 10.1038/s41419-018-1281-7

**Published:** 2019-01-18

**Authors:** Hao Wu, Wei Zhang, Zhenru Wu, Yan Liu, Yujun Shi, Jianping Gong, Wei Shen, Changan Liu

**Affiliations:** 1 Department of Hepatobiliary Surgery, The Second Affiliated Hospital of Chongqing Medical University, Chongqing, 400010 China; 2 Department of Gastroenterology, The Second Affiliated Hospital of Chongqing Medical University, Chongqing, 400010 China; 3 Department of Gastroenterology, The Affiliated Hospital of Guizhou Medical University, Guiyang, 550004 China; 4 Laboratory of Pathology, Key Laboratory of Transplant Engineering and Immunology, NHFPC, West China Hospital, Sichuan University, Chengdu, 610041 China; 5 Department of Gastroenterology, The Fifth people’s Hospital of Chengdu, Sichuan, 611130 China

## Abstract

Accumulating evidence suggests that microRNAs and DNA methylation can cause tumor suppressor gene inactivation and promote tumor malignancy. However, the functional mechanisms of miR-29c-3p and DNA methylation in hepatocellular carcinoma (HCC) are unclear. Here, we reported that miR-29c-3p expression was significantly downregulated in HCC tissues and cell lines. Low miR-29c-3p expression correlated with tumor size, multiplicity pathologic features, and shorter overall survival. Overexpression of miR-29c-3p significantly inhibited HCC cell proliferation, apoptosis, migration, and tumor growth in vivo. Moreover, DNA methyltransferases 3B (DNMT3B) was upregulated in HCC tissues, and was negatively correlated with miR-29c-3p expression. Luciferase reporter and western blotting assays revealed that DNMT3B is a target gene directly regulated by miR-29c-3p. Furthermore, miR-29c-3p regulates the methylation of large tumor suppressor gene 1 (LATS1) by DNMT3B, and abnormal methylation of LATS1 inactivates Hippo signaling pathway. We subsequently identified that high DNMT3B expression and low LATS1 expression were frequently identified in HCC tissues and were associated with poor prognosis. In conclusion, our results indicate that miR-29c-3p acts as a tumor suppressor in HCC by targeting DNMT3B and the LATS1-associated Hippo signaling pathway, which might represent a novel potential therapeutic target for HCC.

## Introduction

Hepatocellular carcinoma (HCC) is the second most frequent cause of cancer-related death, causing 788,000 deaths a year^1^. In recent years, HCC has also become one of the main cancer death causes in China. HCC patients are mostly diagnosed in the middle and late stages wherein intrahepatic and external metastases are often noted. Thus, the prognosis is poor. Effective prevention and treatment measures are currently lacking, and the 5-year recurrence rate after radical resection is up to 61.5%^2^. Tumor recurrence and metastasis remains the main cause of treatment failure for HCC^3,4^. Therefore, it is urgent to clarify the mechanism of recurrence to provide a new strategy for HCC treatment.

MicroRNAs (miRNAs) are a class of single-stranded non-coding small RNA with a length of 21–24 nucleotides. Through incomplete complementarity, miRNAs bind to specific sites of target messenger RNA (mRNA) 3’-non-coding regions, mediating mRNA degradation or inhibiting protein translation. Thus, miRNAs play a key role in mRNA silencing and post- transcriptional expression regulation^5^. More importantly, miRNAs also regulate epigenetic changes by regulating the expression of DNA methyltransferase and directly maintaining DNA methylation^6,7^. Recently, integrated analysis has revealed that the expression of miR-29 family members (29a, 29b, and 29c) in various tumors is negatively correlated with DNA methyltransferase 3A (DNMT3A) and 3B (DNMT3B)^8,9^. MiR-29s directly target 3’-non-coding regions that combine DNMT3A and DNMT3B. In addition, miR-29b regulates DNMT1 expression by downregulating the DNA activator of DNMT1^10^. However, the correlation and the role of miR-29c-3p and DNMT3B in the development of HCC remain unclear.

Large tumor suppressor gene 1 (LATS1), which is the core factor of the Hippo signaling pathway, phosphorylates downstream Yes-associated protein (YAP) and inhibits its ability to act as a transcriptional coactivator^11^. The Hippo signaling pathway regulates the dynamic balance between cell proliferation and apoptosis and effectively controls the development of tissues and organs as well as the generation of tumors^12^. Growing evidence demonstrates the critical role of LATS1 in regulating cell proliferation and the tumor immune response^13^. Emerging evidence indicates that LATS1 exhibits low expression in various human tumors, including gastric cancer^14^, skin cancer^15^, and renal cell carcinoma^16^. However, it is unknown whether LATS1 is involved in the malignant development of HCC via the Hippo signaling pathway.

Many factors are involved in the regulation of LATS1 activity, such as gene mutation, protein phosphorylation, and DNA methylation^17^. DNA methylation can cause inactivation of tumor suppressor genes. Disruption of the methylation of tumor suppressor genes has been observed in HCC. DNA methylation is a chemical modification process catalyzed by DNA methyltransferases (DNMTs), such as DNMT1, DNMT3A, and DNMT3B. In this chemical process, *S*-adenosylmethionine acts as the methyl donor, which provides active methyl to the specific base of the DNA chain. DNA methylation can change the chromatin structure and the accessibility of transcriptional factors, thereby controlling gene expression^18^. DNMT3B is a de novo methyltransferase essential for mammalian development, and DNMT3B expression is increased in many malignancies^19^. Recently, it has been discovered that DNMT3B, in contrast to other methyltransferases, is necessary for gene remethylation and is also a potential therapeutic target to prevent cancer recurrence^20^. However, the relationship between LATS1 methylation and DNMT3B as well as the molecular mechanisms involved remain unclear.

In this study, we demonstrated a significant decrease in miR-29c-3p in HCC tumor tissues and cell lines. Low miR-29c-3p expression in HCC is associated with significantly reduced overall survival (OS) compared with those with high miR-29c-3p expression. Additionally, miR-29c-3p inhibited tumor proliferation, apoptosis, and migration in vitro and tumor growth in vivo. We confirmed that miR-29c-3p activated the Hippo signaling activity through suppression of DNMT3B, and demethylation of LATS1. These findings indicated that miR-29c-3p plays a key role in HCC progression and serves as a potential therapeutic target for HCC.

## Materials and methods

### Patients and tissue samples

In this study, all HCC patient specimens, including hepatocellular carcinoma tumor tissues (T) and paired normal adjacent tissues (N), were retrospectively obtained from May 2007 to July 2012 at the Department of Hepatobiliary Surgery, the Second Affiliated Hospital of Chongqing Medical University, Chongqing, China. All samples were snap-frozen for mRNA and protein assessment. The HCC diagnoses were confirmed histologically by two experienced pathologists in the Department of Pathology Archives of the Second Affiliated Hospital of Chongqing Medical University using hematoxylin and eosin staining. The complete clinical and prognostic data for each tumor tissue sample were recorded, and human tumor tissues used for this research were obtained with informed consent. The study was conducted in accordance with the protocol approved by the Declaration of Helsinki and the guidelines of the Ethics Review Committee of the Second Affiliated Hospital of Chongqing Medical University.

### Cell culture and transfection

In this study, four HCC cell lines, MHCC-97H, HepG2, SMMC-7721, and Huh-7, as well as the LO2 normal liver cell line were obtained from the Institute of Biochemistry and Cell Biology (Chinese Academy of Sciences, Shanghai, China). All cell lines were cultured in high-glucose Dulbecco's modified Eagle's medium (Gibco, USA), whereas the SMMC-7721 and LO2 cell lines were maintained in RPMI-1640 (Gibco, USA). All media were supplemented with 10% fetal bovine serum and 100 units/ml penicillin and streptomycin (HyClone, USA). All cells were grown in a humidified incubator with 5% CO_2_ at 37 °C. Cells were transfected with LV-DNMT3B and LV-NC lentivirus vectors based on the manufacturer’s instruction. Cells were transfected with LATS1 small interfering RNA (siRNA) or negative control (NC) siRNA using Lipofectamine 2000 (Invitrogen) based on the manufacturer’s instruction. Overexpression and silencing of miR-29c-3p were achieved by transfecting cells with pre-miR-29c-3p (miR-29c-3p) and miR-29c-3p inhibitor (anti-miR-29c-3p) lentiviral vectors, respectively. LV-DNMT3B, pre-miR-29c-3p, miR-29c-3p inhibitor, and their associated controls were obtained from GenePharma (Shanghai, China) and the LATS1 siRNA and NC siRNA were obtained from RiboBio (Guangzhou, China).

### Immunohistochemistry

All paraffin-embedded tumor tissues collected from 150 consecutive patients with HCC were used for tissue microarray construction and immunohistochemistry (IHC). The sections were incubated with anti-DNMT3B rabbit polyclonal antibodies (Abcam, USA) and anti-LATS1 rabbit polyclonal antibodies (Abcam, USA) at 1:200 dilution. IHC was performed using the polymer horseradish peroxidase detection system (Zhongshan Goldenbridge Biotechnology, China). The scoring parameters included staining intensity (range 0–3: 0, negative; 1, weak; 2, moderate; and 3, strong) and the percentage of positive cells (range 0–4: 0, negative or <5%; 1, 6%–25%; 2, 26%–50%; 3, 51%–75%; and 4, 76%–100%). We utilized the percentage of positive cells and the intensity to determine the final staining scores. Slides with a total score <4 were defined as having low expression, whereas slides with a score ≥4 were defined as having high expression.

### Quantitative real-time PCR (qRT-PCR)

Total RNA from frozen patient samples and cell lines was extracted using TRIzol reagent (TaKaRa, Japan) according to the manufacturer’s protocol. RNA was reverse transcribed into complementary DNA using PrimeScript RT Reagent (TaKaRa), and qRT-PCR was performed with SYBR Premix Ex Taq II (TaKaRa) using a LightCycler system (Roche). U6 was used as an internal control for microRNA, and GAPDH (glyceraldehyde 3-phosphate dehydrogenase) was used for mRNA. Sequences of the all primers involved are listed in Table S1.

### Bisulfite Sanger sequencing

At least 500 ng of genomic DNA extracted from HCC patient specimens and HCC cell lines was bisulfite converted using a MethylCode™ Bisulfite Conversion Kit (Applied Biosystems, USA). The LATS1 promoter was amplified by PCR with Taq DNA Polymerase (Invitrogen, USA). The primer sequence was designed using Methyl Primer Express™ Software v1.0 (Applied Biosystems, USA). The PCR products were electrophoresed, purified using Spin‑X tubes, and then cloned into the pUC-T vector (both from CWbiotech, Beijing, China). Ten single products were sequenced for each sample.

### DNA extraction and methylation-specific PCR

Genomic DNA was isolated from HCC tumor tissues, paired normal adjacent tissues, and HCC cells using the DNA Isolation kit (Tiangen, Beijing, China) according to the manufacturer’s protocol. Determination of bisulfite conversion was performed using the EpiTect Bisulfite Kit (Qiagen). Methylation-specific PCR (MSP) was performed with 2 μL of bisulfite-modified DNA (100 ng/50 μl) and 48 μL of PCR mixture consisting of 10 × PCR Buffer (Mg2+ free), 25 mM MgCl_2_, dNTP mixture (each 2.5 mM), sense primer (20 μM), antisense primer (20 μM), and TaKaRa EpiTaq HS (5 U/μL; TaKaRa). PCR amplification was conducted using 40 cycles (98 °C for 10 s, 55 °C for 30 s, and 72 °C for 30 s). For parallel quality control, a plasmid containing a methylated LATS1 sequence and water without DNA template were used as positive and negative controls, respectively. Sequences of all methylation primers involved are listed in Table S2.

### Western blot

Proteins from HCC cells and tissues were extracted with RIPA lysis buffer, resolved by sodium dodecyl sulfate polyacrylamide gel electrophoresis, and transferred to a polyvinylidene fluoride membrane (Millipore, USA). The membranes were blocked with 5% non-fat powdered milk at room temperature for 1 h. The membranes were probed at 4 °C overnight with the following specific primary antibodies: DNMT3B (1:1000, Abcam, USA), LATS1 (1:5000, Abcam, USA), p-YAP (1:5000, Abcam, USA), YAP (1:5000, Abcam, USA), BAX (1:5000, Abcam, USA), BCL-2 (1:1000, Abcam, USA), KI67 (1:1000, Abcam, USA), and β-actin (1:5000, Abcam, USA). The membranes were then incubated with the appropriate secondary antibodies. Protein expression levels were visualized using an enhanced chemiluminescence detection system.

### MiRNA in situ hybridization

HCC tissue microarray was treated and hybridized with digoxigenin-labeled miR-29c-3p probes (Exiqon, Copenhagen, Denmark) at 42 °C overnight. Next, the HCC tissue microarray was incubated with mouse anti-digoxin antibody, followed by the streptavidin-biotin-peroxidase complex.

### miRNA target prediction

Four prediction databases, including TargetScan (http://www.targetscan.org), Oncomir (http://www.oncomir.org/), MiRanda (http://www.microrna.org/microrna/home.do) and miRWalk (http://mirwalk.umm.uni-heidelberg.de/), were used to predict miRNA targets and conserved sites bound by miR-29c-3p.

### Flow cytometry analysis of cell apoptosis

HCC cell apoptosis was quantified using a FITC-labeled Annexin V/propidium iodide (PI) Apoptosis Detection kit (Beyotime, China) according to the instructions. Flow cytometric analysis was performed immediately after staining using a flow cytometer (Beckman, USA).

### Wound healing assay

Briefly, 5 × 10^5^ HepG2 and MHCC-97H cells were grown in six-well plates. After reaching confluence, non-adherent cells were washed away twice with phosphate-buffered saline (PBS). The cell monolayer was scratched with a pipette tip (10 ml) to generate 3 scratch wounds and then rinsed twice with PBS to remove non-adherent cells. After 0, 24 and 48 h, the distance between the wound sides was measured.

### 5-Ethynyl-2′-deoxyuridine (EdU) assay

Cells were seeded into 96-well plates (5 × 10^3^ cells/well) and cultured for 24 h. Cells were incubated with EdU (50 μM) for 2 h at 37 °C, fixed in 4% formaldehyde for 30 min, and permeabilized with 0.5% Triton X-100 solution for 10 min at room temperature. After washing with PBS, 1× ApolloR reaction cocktail (100 μL) was added, and the reaction proceeded for 30 min at room temperature in the dark. Cells nuclei were stained by adding 1× Hoechst 33342 (100 μL) for 30 min. Cell proliferation was analyzed using the mean number cells in three fields for each sample.

### Luciferase reporter assay

The wild-type DNMT3B-3′-untranslated region (UTR) (WT) and mutant DNMT3B-3’-UTR (MUT) containing the putative binding site of miR-29c-3p were cloned and established in the Firefly luciferase-expressing pMIR-REPORT vector (Obio Technology, China). HCC cells were seeded into 24-well plates for 24 h and then transfected with pMIR-REPORT-DNMT3B-3’-UTR-WT or the pMIR-REPORT-DNMT3B-3’-UTR-MUT reporter vector. Both vectors were transfected using Lipofectamine 2000 (Invitrogen). After 48  h, cells were collected, and luciferase assays were performed using the Luciferase Reporter Assay System (GloMax) according to the manufacturer’s protocol.

### Cell-counting kit-8 assay

The cell-counting kit-8 assay (CCK-8, Dojindo, Japan) was used to analyze cell proliferation according to the manufacturer’s instructions. For the CCK-8 assay, cells were seeded at 2000 cells/well in 96-well plates. After 24  h, 10 ul of CCK-8 was added to each well. The absorbance at 450  nm was recorded for each well to assess cell proliferation.

### Colony-forming assay

For the colony-forming assay, HCC cells were plated in 6-well plates (500 cells/well) containing 2.5 mL of medium and cultured for 3 weeks. Colonies formed by cell proliferation were stained with crystal violet, and colonies containing at least 50 cells were counted. Colonies were fixed with 20% methanol and stained with 0.1% crystal violet, and the colonies were counted.

### Immunofluorescence of cells

Cells were seeded and cultured on coverslips. After the corresponding treatment, cells were fixed with 4% paraformaldehyde and permeabilized with 0.25% Triton X-100 solution for 20 min. Cells were washed with PBS and then blocked in 5% bovine serum albumin for 1 h at room temperature. Coverslips were incubated with DNMT3B (1:500, Abcam, USA) and LATS1 (1:500, Abcam, USA) antibodies overnight at 4 °C. After washing with PBS, cells were incubated with appropriate secondary antibody and 4′,6-diamidino-2-phenylindole (DAPI). Slides were imaged using an inverted fluorescence microscope, and results were recorded.

### Mouse xenograft tumor model

The 5-week-old male BALB/c-nu mice were purchased from Shanghai Experimental Animal Center (Shanghai, China) and fed in the Experimental Animal Center of Chongqing Medical University (Chongqing, China). All animal experiments were performed in accordance with the institutional guidelines. For the cell proliferation study in vivo, miR-29c-3p and their associated negative control MHCC97-H and HepG2 cells (5 × 10^6^) were subcutaneously injected into the left hip flanks of the mice. Tumor growth was recorded once a week with calliper measurements. Tumor volume was calculated according to the following formula: volume = (width^2^ × length)/2. At 50 days after injection, the mice were killed, and tumors were collected for analysis.

### Statistical analysis

All data were analyzed using SPSS 20.0 software (SPSS Inc., Chicago, IL, USA) or GraphPad Prism version 6.0 (CA, USA). Data were presented as the means ± standard deviation (SD). Statistical differences were analyzed by Student’s *t*-test, χ^2^ test, and repeated measures analysis of variance. The correlation between miR-29c-3p and DNMT3B mRNA expression was evaluated using Spearman’s correlation analysis. The Kaplan–Meier method was used to assess OS, and the log-rank test was used to analyze the differences between the curves. The prognostic meaning of miR-29c-3p, DNMT3B, and LATS1 expression was calculated by univariate and multivariate Cox regression analysis. The threshold for statistical significance was *p* < 0.05.

## Results

### MiR-29c-3p is downregulated in HCC, and is significantly correlated with poor clinical prognosis

**Fig. 1:**
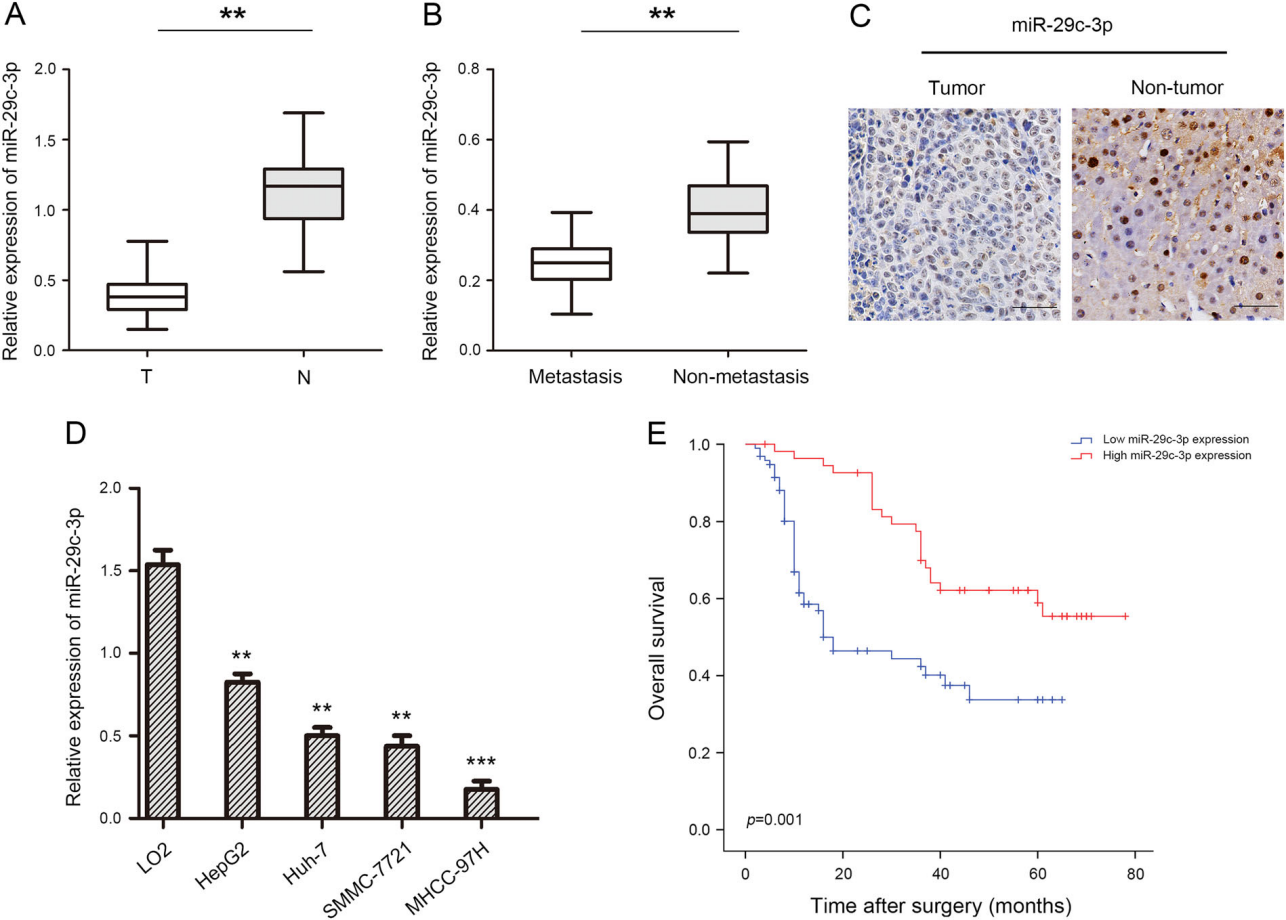
miR-29c-3p is downregulated in human hepatocellular carcinoma (HCC) samples. **a** Relative expression of miR-29c-3p in 150 pairs of HCC tissues and paired normal adjacent tissues. **b** Relative expression of miR-29c-3p expression in aggressive and nonaggressive HCC tissues. **c** miR-29c-3p expression in HCC tissues and paired normal adjacent tissues. **d** Relative miR-29c-3p expression in 4 HCC cell lines, and the normal human hepatocyte cell line LO2 served as the control. **e** Kaplan–Meier analysis of overall survival between high (*n* = 55) and low (*n* = 95) miR-29c-3p expression in HCC patients; **p < 0.01, ***p < 0.001

To explore the role of miR-29c-3p in HCC, we performed qRT-PCR to detect miR-29c-3p levels in HCC tissues and paired normal adjacent tissues. We found that miR-29c-3p expression was obviously downregulated in 63.3% (95 of 150) HCC tissues compared with paired normal adjacent tissues (Fig. 1a). Moreover, miR-29c-3p expression was significantly reduced in aggressive HCC tissues compared with nonaggressive HCC tissues (Fig. 1b). Next, miRNA in situ hybridization revealed that miR-29c-3p was more highly expressed in paired normal adjacent tissues compared with HCC tissues (Fig. 1c). Similarly, in accordance with miR-29c-3p expression in HCC tissues, miR-29c-3p expression was also decreased in HCC cell lines with different metastatic abilities compared with the normal human hepatocyte line LO2 (Fig. 1d). Importantly, miR-29c-3p expression was reduced in HCC cell lines that exhibited strong metastatic ability.

Interestingly, miR-29c-3p expression was positively correlated with tumor size (*p* = 0.018), multiplicity (*p* = 0.011), and intrahepatic metastasis (*p* = 0.035) (Table 1). In addition, Kaplan–Meier survival analysis revealed that low miR-29c-3p expression with HCC exhibited significantly shorter OS compared with those with high miR-29c-3p expression (Fig. 1e). In univariate analysis, miR-29c-3p (*p* *=* 0.002) and tumor size (*p* *=* 0.031) were significantly associated with OS. The multivariate model revealed that OS was significantly dependent on miR-29c-3p (*p* *=* 0.015) and tumor size (*p* *=* 0.008) (Table 2), suggesting that miR-29c-3p was an independent prognostic factor for OS with HCC. These results demonstrate that miR-29c-3p may be involved in the malignant progression of HCC.

**Table 1:** Correlations between miR-29c-3p with clinicopathological features of HCC patients

Variables	*n*	miR-29c-3p expression		*P*
		Low (*n* = 95)	High (*n* = 55)	
Age (years)				
<50	82	53	29	0.736
≥50	68	42	26	
Gender				
Male	92	57	35	0.659
Female	58	38	20	
Tumor size (cm)				
≤5	76	41	35	**0.018**
>5	74	54	20	
AFP (ng/mL)				
≤20	69	48	21	0.175
>20	81	47	34	
Liver cirrhosis				
Presence	85	55	30	0.734
Absence	65	40	25	
HBsAg				
Positive	94	61	33	0.726
Negative	56	34	22	
TNM stage				
I/II	91	53	38	0.120
III/IV	59	42	17	
Vascular invasion				
Presence	74	43	31	0.236
Absence	76	52	24	
Multiplicity				
Single	71	37	34	**0.011**
Multiple (≥2)	79	58	21	
Intrahepatic metastasis				
Presence	85	60	25	**0.035**
Absence	65	35	30	

*HCC* hepatocellular carcinoma, *AFP* alpha-fetoprotein, *HBsAg* hepatitis B surface antigen,*TNM* tumor, node, metastasis

Bold values indicate statistical significance

**Table 2:** Univariate and multivariate analysis of different prognostic variables and overall survival (OS) in HCC patients

Variables	*n*	Univariate analysis		Multivariate analysis model	
		HR (95% CI)	*P*	HR (95% CI)	*P*
Sex		1.369 (0.843–1.695)	0.869		
Female	58				
Male	92				
Age (year)		2.016 (0.860–1.961)	0.692		
<50	82				
≥50	68				
AFP (ng/mL)		1.654 (0.598–1.693)	0.581		
≤20	69				
>20	81				
HBsAg		2.463 (1.596–2.032)	0.439		
Positive		94			
Negative		56			
Liver cirrhosis		0.961 (0.536–1.934)	0.506		
Presence	85				
Absence	65				
TNM stage		0.933 (0.536–1.964)	0.783		
I/II	91				
III/IV	59				
Tumor size (cm)		1.862 (1.639–2.533)	**0.031**	0.869 (1.036–2.695)	**0.008**
≤5	76				
>5	74				
Multiplicity		0.969 (0.569–1.965)	0.736		
Single	71				
Multiple (≥2)	79				
Intrahepatic metastasis		0.693 (0.669–1.546)	0.963		
Presence	85				
Absence	65				
Vascular invasion		1.236 (1.893–2.361)	0.901		
Presence	74				
Absence	76				
miR-29c-3p expression		2.369 (1.069–2.465)	**0.002**	2.023 (1.530–3.039)	**0.015**
Low	95				
High	55				

*HCC* hepatocellular carcinoma, *HR* hazard rate, *CI* confidence interval, *AFP* alpha-fetoprotein, *HBsAg* hepatitis B surface antigen,*TNM* tumor, node, metastasis

Bold values indicate statistical significance

### Increased miR-29c-3p inhibits HCC cell proliferation, migration, and invasion and induces HCC cell apoptosis in vitro

**Fig. 2:**
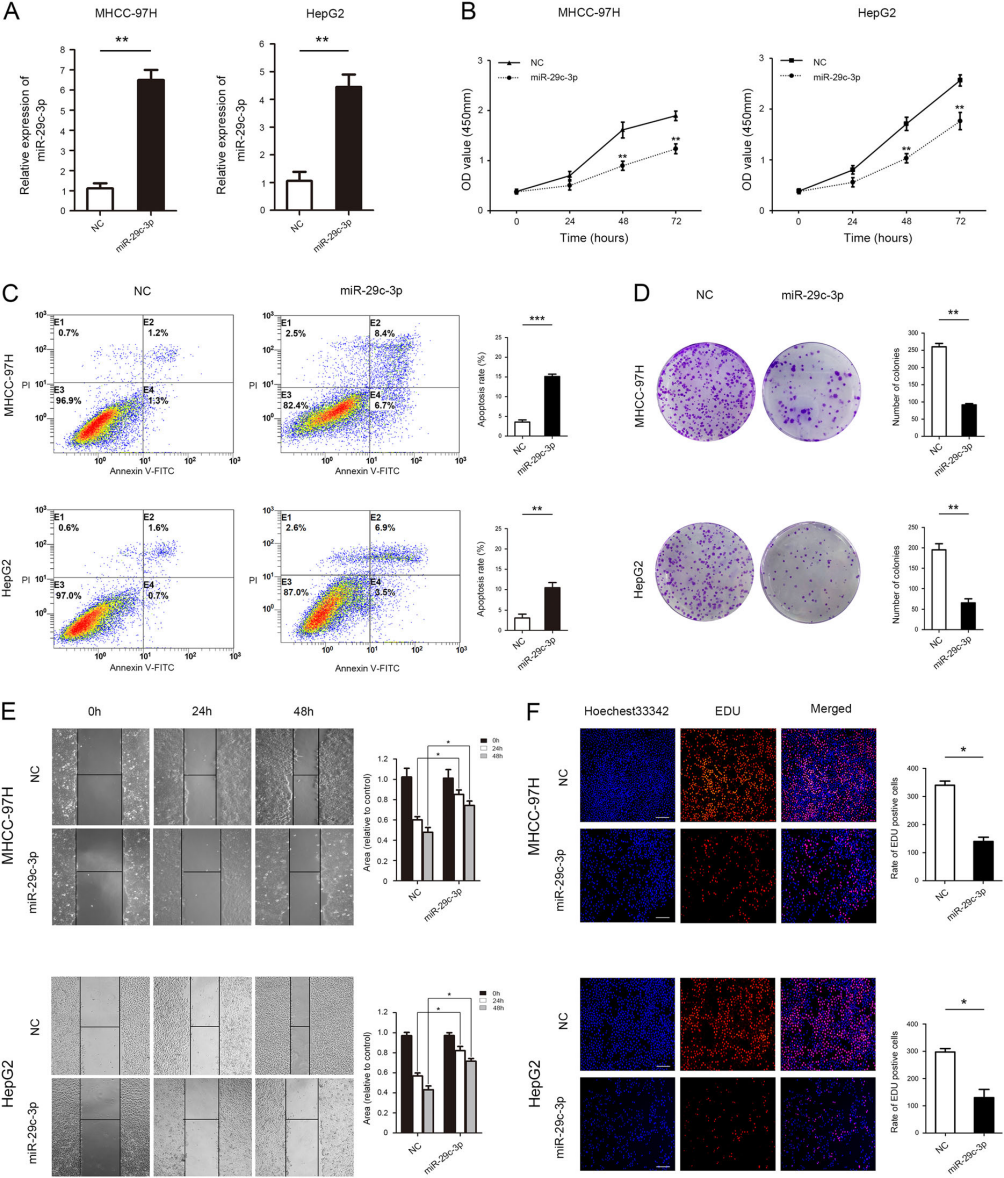
Increased miR-29c-3p inhibits hepatocellular carcinoma (HCC) cell proliferation and migration and induces HCC cell apoptosis in vitro. **a** MHCC-97H and HepG2 cells were transfected with pre-miR-29c-3p, and miR-29c-3p expression was measured by quantitative real-time PCR (qRT-PCR). **b** Effect of miR-29c-3p on HCC cell proliferation was analyzed using CCK-8 assays. **c** Flow cytometry was used to analyze the effect of miR-29c-3p on HCC cell apoptosis. **d** Colony formation assays in HCC cells after overexpression of miR-29c-3p. **e** Wound healing assays were performed to determine the effects of miR-29c-3p overexpression on HCC cell migrations. **f** Cell proliferation was detected with 5-ethynyl-2′-deoxyuridine (EdU) in HCC cells after miR-29c-3p overexpression; *p < 0.05, **p < 0.01, ***p < 0.001

To further investigate the effects of miR-29c-3p on HCC malignancy, both gain-of-function experiments were performed in MHCC-97H and HepG2 cell lines, which exhibited different levels of miR-29c-3p. Using qRT-PCR, we confirmed that miR-29c-3p was effectively overexpressed in both cell lines (Fig. 2a). CCK-8 assays revealed that increase in miR-29c-3p expression significantly inhibited cell proliferation in MHCC-97H and HepG2 cells compared with those in the NC groups (Fig. 2b). Fluorescein isothiocyanate (FITC)-conjugated Annexin V and PI staining was then used to measure the effect of miR-29c-3p on apoptosis. The results indicate that miR-29c-3p significantly induced apoptosis in MHCC-97H and HepG2 cells (Fig. 2c). In addition, colony formation assays revealed that miR-29c-3p overexpression remarkably decreased colony formation abilities in MHCC-97H and HepG2 cell (Fig. 2d). MHCC-97H and HepG2 cell mobility in wound healing assays significantly decreased with miR-29c-3p overexpression (Fig. 2e). As shown in Fig. 2f, the number of HCC cells incorporating EdU in the miR-29c-3p overexpression group was less than the control group.

### MiR-29c-3p activates Hippo signaling to suppress tumorigenicity in HCC

**Fig. 3:**
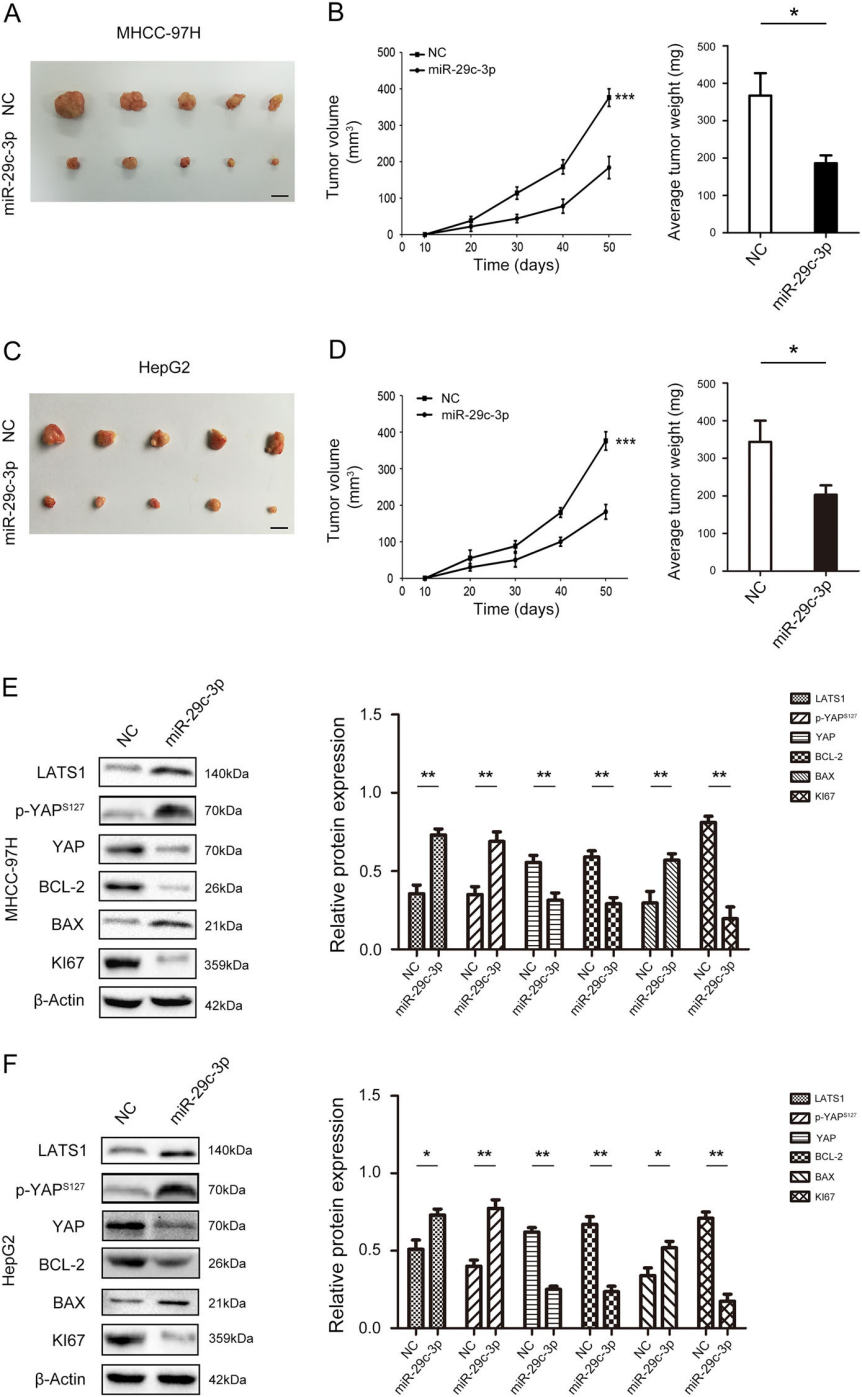
miR-29c-3p inhibits tumorigenicity of hepatocellular carcinoma (HCC) in vivo and activates Hippo signaling. **a** MHCC-97H cells transfected with pre-miR-29c-3p were subcutaneously inoculated into nude mice to form tumor. **b** Tumor volume and weight growth curves were recorded. **c** HepG2 cells transfected with pre-miR-29c-3p were subcutaneously inoculated into nude mice to form tumors. **d** Tumor volume and weight growth curves were recorded. **e** Protein expression of Hippo signaling pathway components, including proliferation- and apoptosis-related indicators, after transfection with pre-miR-29c-3p in MHCC-97H cells. **f** Protein expression of Hippo signaling pathway components, including proliferation- and apoptosis-related indicators, after transfection with pre-miR-29c-3p in HepG2 cells; *p < 0.05, **p < 0.01, ***p < 0.001

Based on our results in vitro, we further identified the influence of miR-29c-3p on proliferation in vivo, MHCC-97H and HepG2 cells were stably transfected with miR-29c-3p overexpression vector or the NC vector and then injected into the flanks of nude mice to form subcutaneous ectopic tumors. As shown in Fig. 3a, b, the tumor volume and weight in the miR-29c-3p overexpressed group were much smaller compared with the control transfected group in MHCC-97H cells. Similar effects of miR-29c-3p were observed in the HepG2 cell model (Fig. 3c, d). The Hippo signaling pathway regulates cell proliferation and apoptosis to control tissue size^11^, and our results demonstrated that miR-29c-3p overexpression activated the Hippo signaling pathway and inhibited YAP expression, resulting in decreased Bcl-2 and Ki67 expression and increased Bax expression (Fig. 3e). Similar effects of miR-29c-3p were observed in the HepG2 cell model (Fig. 3f). Therefore, these results indicate that overexpression of miR-29c-3p suppresses tumor growth of HCC via Hippo signaling.

### Hypermethylation of LATS1 promoter CpG island contributes to LATS1 silencing in HCC

**Fig. 4:**
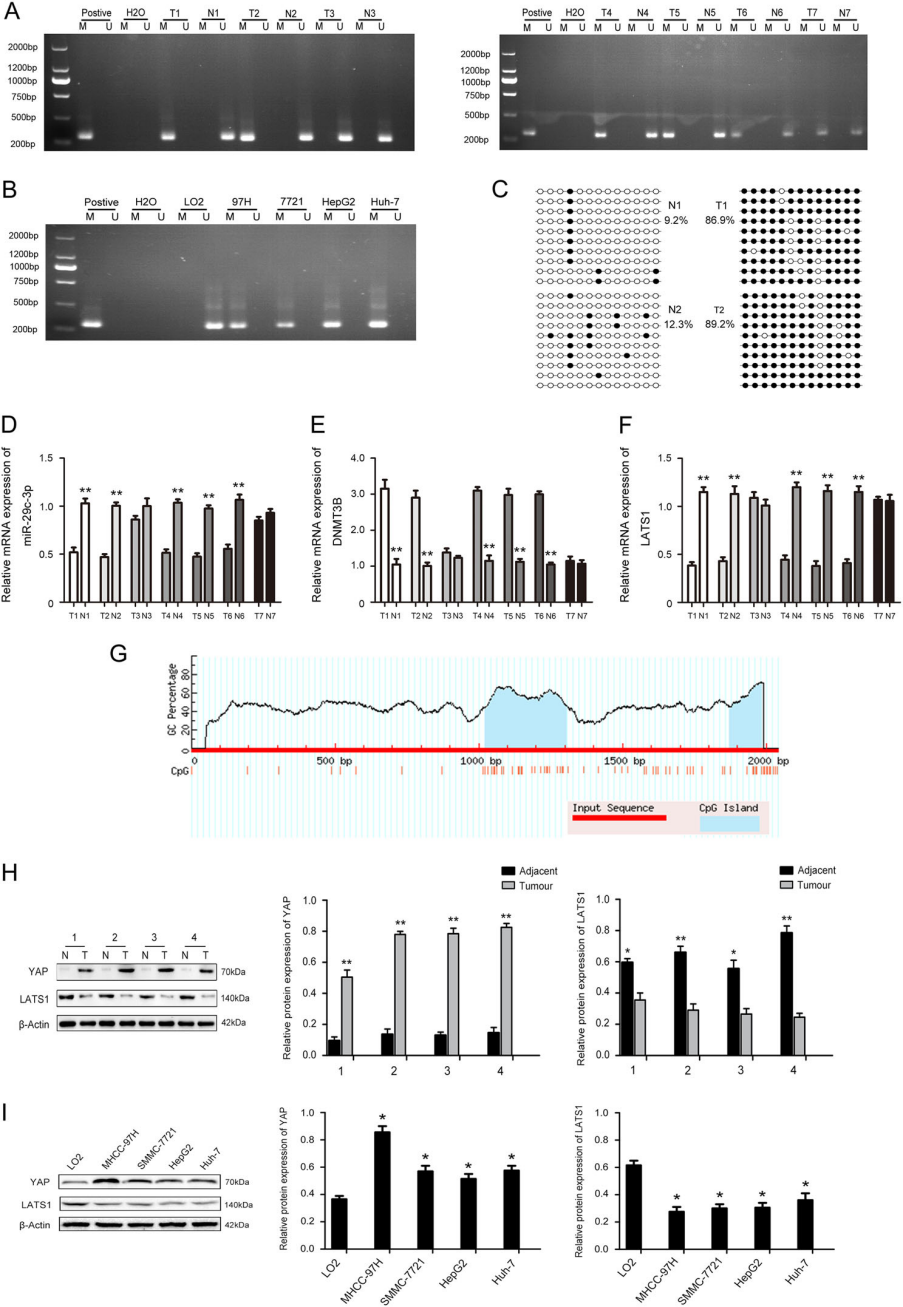
Aberrant DNA hypermethylation and expression of large tumor suppressor gene 1 (LATS1) in hepatocellular carcinoma (HCC) and HCC cell lines. **a** The methylation status of LATS1 was randomly detected in 7 HCC and paired normal adjacent tissues. **b** The methylation status of LATS1 was detected in LO2, MHCC-97H, HepG2, SMMC-7721, and Huh7cell lines. **c** Bisulfite sequencing analysis was performed on LATS1 promoter methylation in HCC tissues compared with paired normal adjacent tissues. **d** The relative mRNA expression of miR-29c-3p in 7 HCC and paired normal adjacent tissues. **e** The relative mRNA expression of DNA methyltransferase 3B (DNMT3B) in 7 HCC and paired normal adjacent tissues. **f** The relative mRNA expression of LATS1 in 7 HCC and paired normal adjacent tissues. **g** The CpG islands (shaded area) of LATS1 promoter region. **h** YAP and LATS1 protein expression in HCC and paired normal adjacent tissues. **i** YAP and LATS1 protein expression in HCC and LO2 cells; *p < 0.05, **p < 0.01. Black dots, methylation, white dots, unmethylated

LATS1 is methylated and exhibits low expression, resulting in inactivation of the Hippo signaling pathway^12,15^. The methylation status of LATS1 was assayed in 7 randomly selected HCC tissues compared with paired adjacent normal tissues. The results displayed that LATS1 hypermethylation was frequently observed in 5 (71.4%) of 7 HCC compared with paired adjacent normal tissues (Fig. 4a). Analysis of LO2, MHCC-97H, HepG2, SMMC-7721, and Huh-7 revealed a significantly high degree of methylation in HCC cell line, but not in LO2 cells (Fig. 4b). We assessed the exact methylation sites and the extent of CpG methylation of LATS1 by bisulfite sequence‑PCR (BSP). LATS1 promoter methylation in HCC tissues was increased compared with paired adjacent normal tissues (Fig. 4c), and the exact methylation sites are indicated in Fig. 4g.

Furthermore, we found low expression of miR-29c-3p and LATS1 and high expression of DNMT3B in LATS1-methylated HCC tissues by qPCR (Fig. 4d–f). Next, we randomly detected LATS1 and YAP expression in four HCC tissues and paired normal adjacent tissues. LATS1 and YAP expression levels in tumor tissues were downregulated and upregulated, respectively (Fig. 4h). As shown in Fig. 4i, the same results were also observed in HCC cells. These results strongly suggest that LATS1 undergoes hypermethylation in HCC, which inactivates the Hippo signaling pathway.

### MiR-29c-3p directly interacts with DNMT3B

**Fig. 5:**
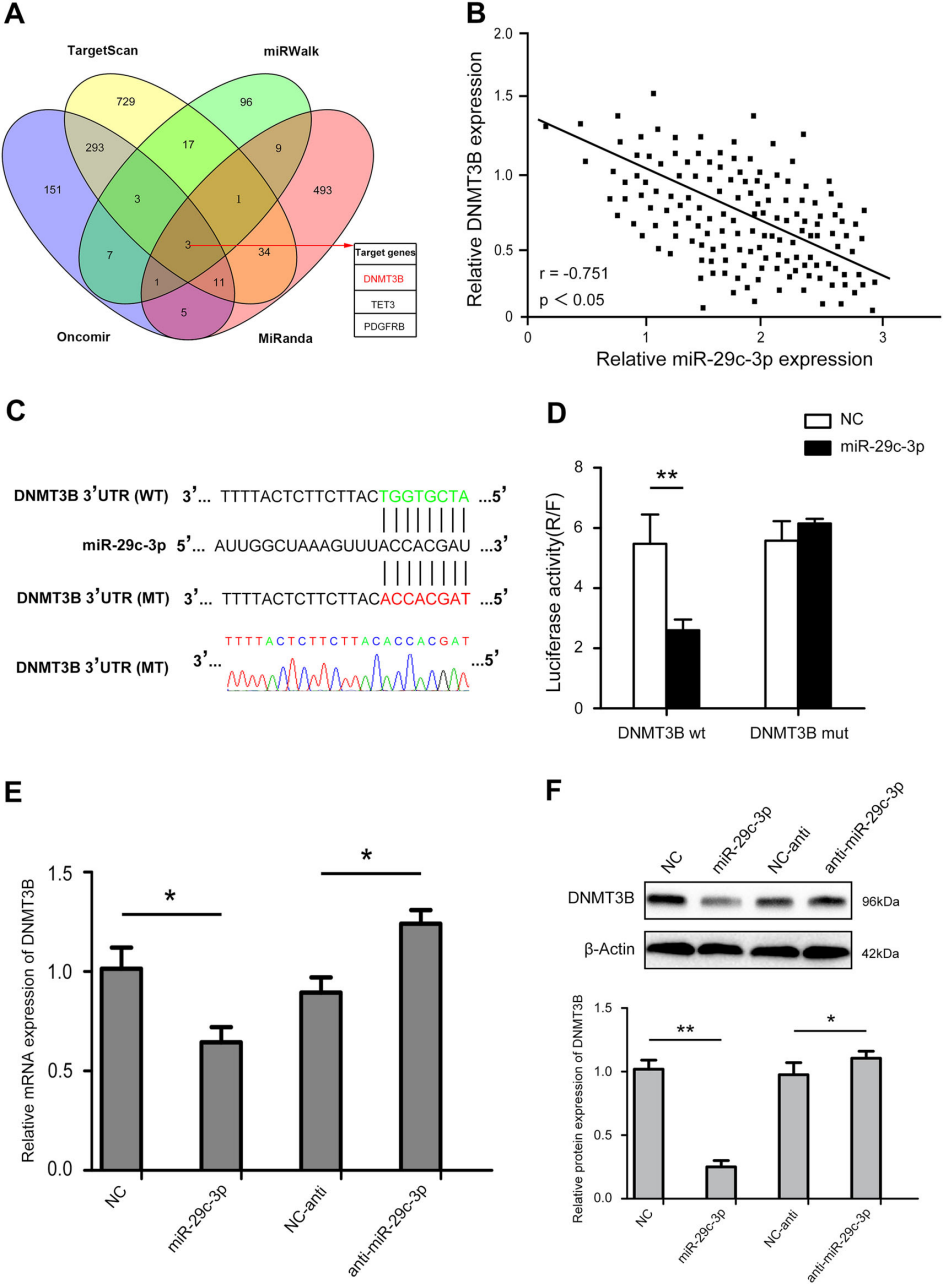
miR-29c-3p directly targets DNA methyltransferase 3B (DNMT3B) and miR-29c-3p levels were inversely correlated with DNMT3B protein levels. **a** Venn diagram displaying miR-29c-3p computationally predicted to target DNMT3B by four different prediction algorithms: TargetScan, MiRanda, Oncomir, and miRWalk. **b** miR-29c-3p expression was negatively correlated with DNMT3B expression in hepatocellular carcinoma (HCC) tissues. Spearman's rank test (*r* = −0.751, *p* < 0.05). **c** Diagrams reveal putative miR-29c-3p binding sites and corresponding mutant sites of DNMT3B. **d** The luciferase activity of each combination was assessed. **e** DNMT3B mRNA expression in MHCC-97H-pre-miR-29c-3p cells and MHCC-97H -miR-29c-3p inhibitor cells. **f** Western blotting was performed after transfecting cells with pre-miR-29c-3p or miR-29c-3p inhibitors in MHCC-97H; *p < 0.05, **p < 0.01

DNMT3B plays a major role in the de novo methylation of genes^18^. We sought to further investigate the underlying molecular mechanisms by which miR-29c-3p exerts its functional effects on HCC cells. We examined miRNA databases, including TargetScan, miRWalk, Oncomir and miRanda, and identified that DNMT3B was listed as a potential target of miR-29c-3p (Fig. 5a). Our results demonstrated that miR-29c-3p expression was inversely associated with DNMT3B expression (Fig. 5b). The complementary sequence of miR-29c-3p was found in the 3’-UTR of DNMT3B mRNA (Fig. 5c). Next, we generated luciferase reporter vectors containing the wild-type (Wt) or mutant (Mt) DNMT3B 3’-UTR sequences to reveal the interaction of miR-29c-3p with DNMT3B. Co-transfection of miR-29c-3p significantly inhibited luciferase activity in cells transfected with Wt DNMT3B 3’-UTR. In contrast, the inhibition was not observed in cells co-transfected with Mt DNMT3B 3’-UTR (Fig. 5d), indicating that miR-29c-3p directly targets DNMT3B. As shown in Fig. 5e, f, qRT-PCR and western blot assays revealed that miR-29c-3p overexpression significantly reduced DNMT3B mRNA and protein levels. Taken together, these results suggest that DNMT3B is a direct target of miR-29c-3p.

### Overexpression of miR-29c-3p increases LATS1 expression and demethylation of LATS1 via DNMT3B

**Fig. 6:**
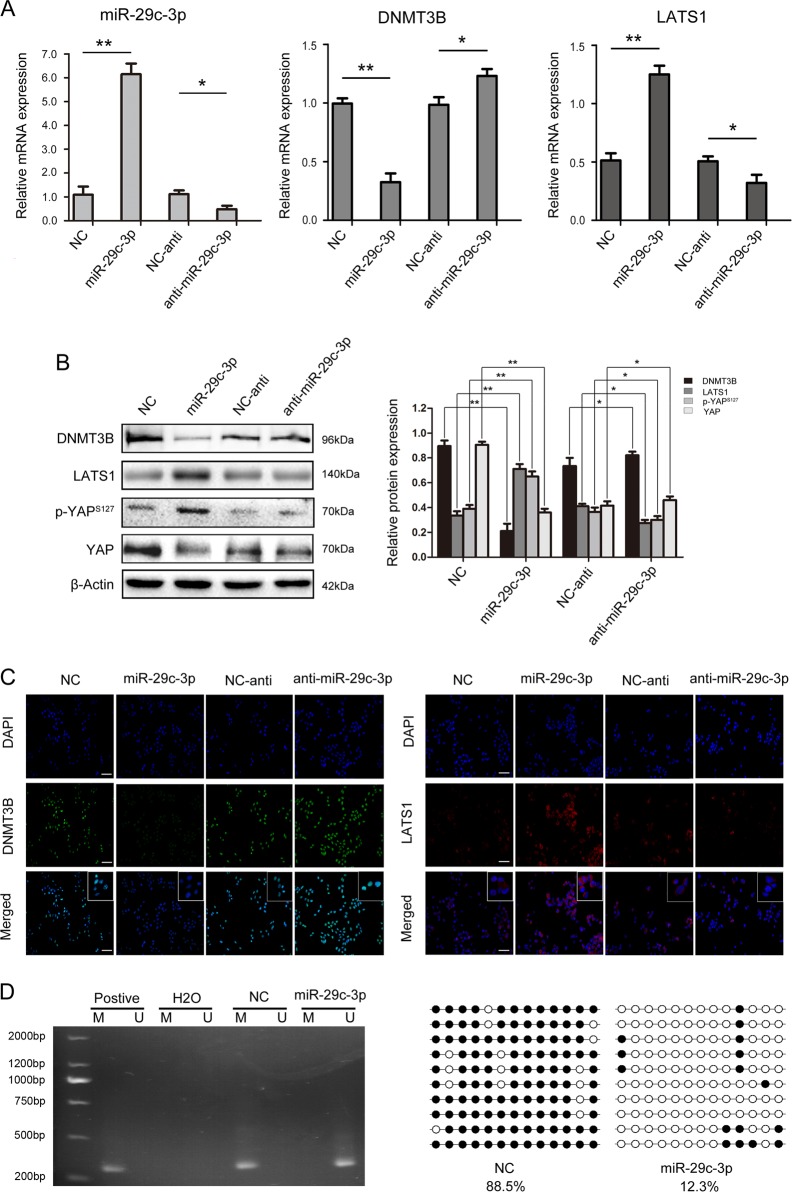
miR-29c-3p regulates the expression of large tumor suppressor gene 1 (LATS1) via DNA methyltransferase 3B (DNMT3B). **a** mRNA expression levels of miR-29c-3p, DNMT3B and LATS1 after transfection with pre-miR-29c-3p and miR-29c-3p inhibitor in MHCC-97H cells. **b** After transfection with pre-miR-29c-3p and miR-29c-3p inhibitor, DNMT3B, LATS1, p-YAP^s127^, and YAP expression levels were detected by western blot. **c** After transfection with pre-miR-29c-3p and miR-29c-3p inhibitor, DNMT3B and LATS1 expression in MHCC-97H cells was detected by immunofluorescence. **d** The methylation status of LATS1 after transfection with pre-miR-29c-3p in MHCC-97H cells was detected by methylation-specific PCR (MSP) and bisulfite sequence‑PCR (BSP); *p < 0.05, **p < 0.01. Black dots, methylation, white dots, unmethylated

To examine whether miR-29c-3p regulates LATS1 expression via DNMT3B, we overexpressed and repressed miR-29c-3p in MHCC-97H cells. Forced expression of miR-29c-3p significantly reduced DNMT3B mRNA expression and increased LATS1 mRNA expression (Fig. 6a). Correspondently, DNMT3B and LATS1 protein levels were significantly reduced and increased, respectively, and YAP expression was decreased, which is downstream of LATS1, these effects resulted in Hippo signaling pathway activation (Fig. 6b, c). More importantly, overexpression of miR-29c-3p resulted in demethylation of the LATS1 gene (Fig. 6d). Interestingly, silencing LATS1 rescues the effect of overexpression of miR-29c-3p on HCC progression (Supplementary Fig. 1). Collectively, these results indicated that miR-29c-3p downregulates DNMT3B, which subsequently leads to the re-activation of LATS1 through the demethylation of LATS1.

### DNMT3B ameliorates the inhibitory effect of miR-29c-3p on HCC progression

**Fig. 7:**
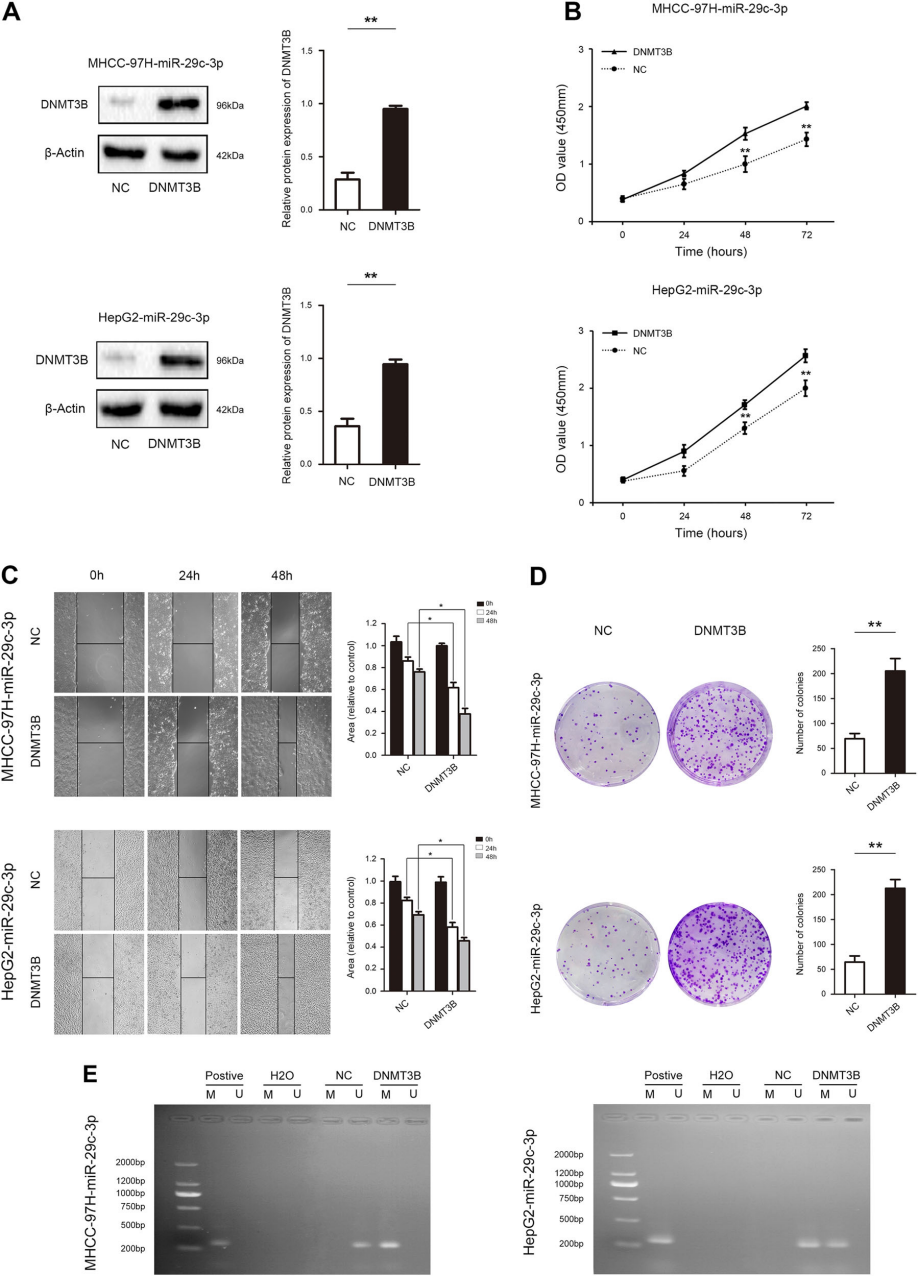

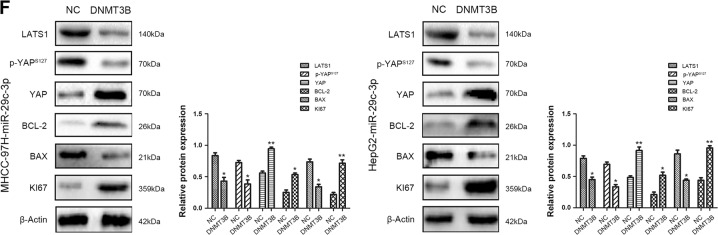
Rescue experiments are performed to confirm that DNA methyltransferase 3B (DNMT3B) is the functional target of miR-29c-3p in hepatocellular carcinoma (HCC) progression. **a** Western blot revealed DNMT3B protein expression in MHCC-97H-miR-29c-3p cells and HepG2-miR-29c-3p that were transfected with DNMT3B vector and NC. **b** Proliferation of MHCC-97H-miR-29c-3p cells and HepG2-miR-29c-3p cells that were transfected with DNMT3B vector and negative control (NC) was determined by CCK-8 assay. **c** Wound healing assay was performed to determine the effects of DNMT3B on HCC cell migration. **d** Colony formation assays assessed the effects of DNMT3B on HCC cell proliferation. **e** The methylation status of large tumor suppressor gene 1 (LATS1) was detected in MHCC-97H-miR-29c-3p cells and HepG2-miR-29c-3p cells that were transfected with DNMT3B vector and NC. **f** Western blot revealed the expression of Hippo signaling pathway components, including proliferation- and apoptosis-related indicators in MHCC-97H-miR-29c-3p cells and HepG2-miR-29c-3p cells that were transfected with DNMT3B vector and NC; *p *<* 0.05, **p *<* 0.01

Next, rescue experiments were performed to confirm whether miR-29c-3p executed its functional effects by suppressing its target genes. DNMT3B expression was restored after miR-29c-3p overexpression in MHCC-97H and HepG2 cells (Fig. 7a). Restoration of DNMT3B expression significantly abolished the inhibitory effects of miR-29c-3p on proliferation and migration (Fig. 7b–d). Importantly, reintroduction of DNMT3B causes remethylation of LATS1 and inactivation of the Hippo signaling pathway, resulting in the expression of index proteins that inhibit apoptosis and promote proliferation in HCC (Fig. 7e, f).

### DNMT3B is upregulated and LATS1 is downregulated in HCC

**Fig. 8:**
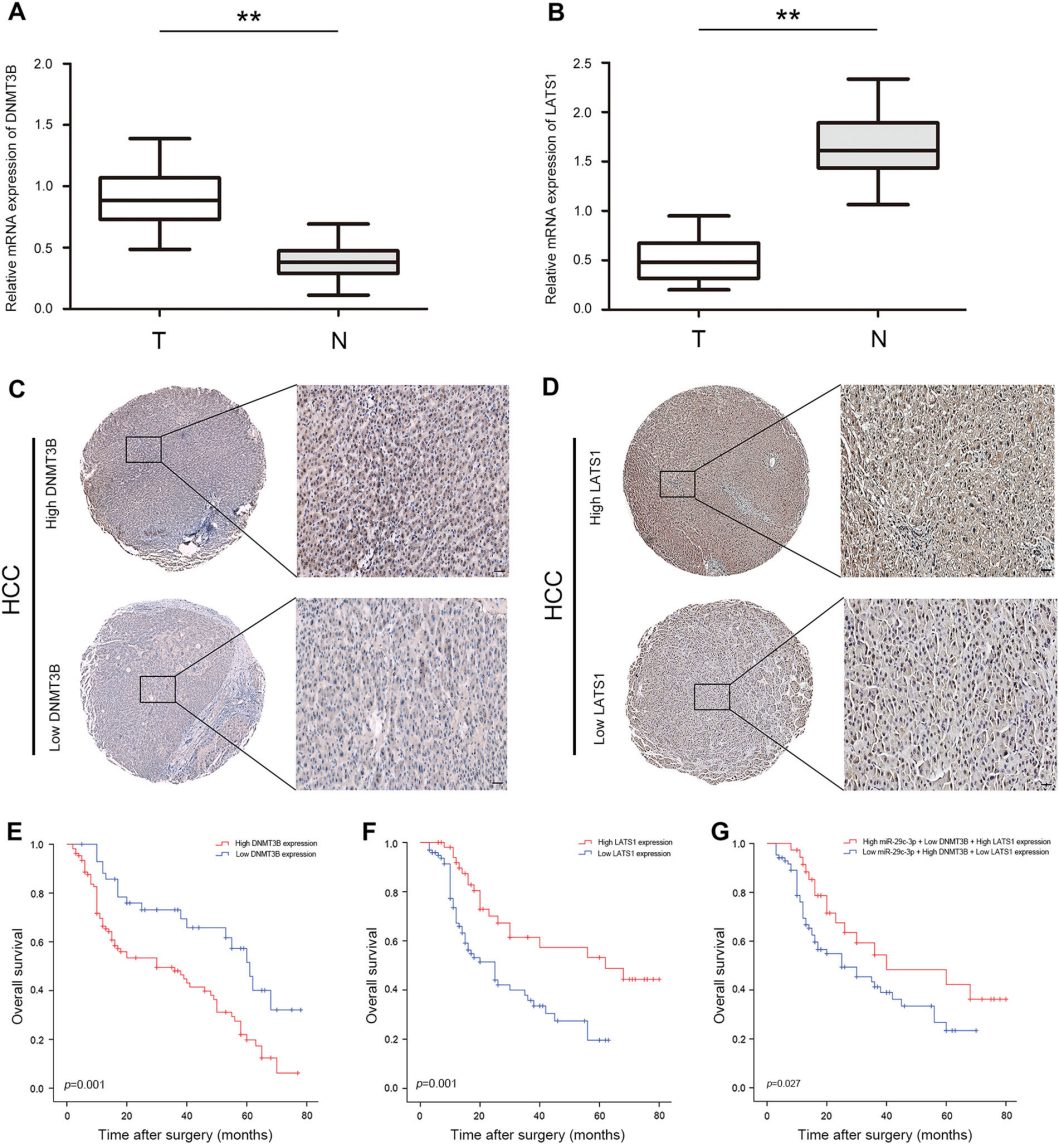
DNA methyltransferase 3B (DNMT3B) is upregulated and large tumor suppressor gene 1 (LATS1) is downregulated in hepatocellular carcinoma (HCC). **a** Quantitative real-time PCR (qRT-PCR) analysis of DNMT3B expression in 150 pairs of HCC tissues and paired normal adjacent tissues. **b** qRT-PCR analysis of LATS1 expression in 150 pairs of HCC tissues and paired normal adjacent tissues. **c** Immunohistochemical staining analysis of DNMT3B protein expression levels in HCC tissues. **d** Immunohistochemical staining analysis of LATS1 protein expression levels in HCC tissues. **e** Kaplan–Meier analysis of overall survival between HCC patients with high and low DNMT3B expression. **f** Kaplan–Meier analysis of overall survival between high and low LATS1 expression in HCC patients. **g** Kaplan–Meier analysis of overall survival between the high miR-29c-3p/low DNMT3B/high LATS1 expression group and low miR-29c-3p/high DNMT3B/low LATS1 expression group; **p < 0.01

To further investigate the role of DNMT3B and LATS1 in HCC, DNMT3B and LATS1 mRNA levels were also validated by qRT-PCR in the previous cohort of 150 paired HCC clinical specimens. DNMT3B mRNA level was significantly upregulated (107 of 150,71.3%) and LATS1 was significantly downregulated (97 of 150,64.6%) in HCC tissues (Fig. 8a, b). Additionally, DNMT3B and LATS1 expression in HCC tissues was detected by immunohistochemical staining analysis, and the results indicate dramatically increased DNMT3B expression and decreased LATS1 expression in HCC, respectively (Fig. 8c, d). As shown in Fig. 8e, high DNMT3B expression in HCC was associated with significantly reduced OS compared with those with low DNMT3B expression (*p* = 0.001). Low LATS1 expression in HCC was associated with significantly reduced OS compared with those with high LATS1 expression (*p* *=* 0.001) (Fig. 8f). Moreover, low miR-29c-3p/high DNMT3B/low LATS1 expression in HCC was associated with significantly shorter OS compared with those with high miR-29c-3p/low DNMT3B/high LATS1 expression (*p* = 0.027) (Fig. 8g). Next, DNMT3B expression was positively correlated with tumor size, vascular invasion, and intrahepatic metastasis (*p* < 0.005), and LATS1 expression was positively correlated with tumor size, TNM (tumor, node, metastasis) stage, and intrahepatic metastasis (*p* *<* 0.005) (Table S3). Multivariate Cox regression analyses further demonstrate that DNMT1B and LATS1 were independent prognostic factor of HCC (Tables S4, S5).

## Discussion

As a malignant tumor, HCC exhibits a higher rate of recurrence and proliferation. Patients often have poor prognosis, causing a huge burden on families and society^1^. Therefore, it is urgent to identify an effective target for therapy. The purpose of our study was to clarify the underlying regulatory mechanism of miR-29c-3p in HCC and the mechanism by which miR-29c-3p inhibits the malignant progression of HCC. In this study, we discovered that miR-29c-3p is lowly expressed in HCC, and miR-29c-3p directly regulates DNMT3B-mediated methylation of LATS1. Then, we altered the expression of LATS1 and Hippo signaling pathway activation to inhibit the malignant progression of HCC. In addition, recent studies have indicated that miR-29c-3p may function as a biomarker for cancers^21^.

Next, we found that upregulation of miR-29c-3p inhibits proliferation and migration and promotes apoptosis as well as tumor growth in vivo, and Hippo signaling pathway was inactive in HCC. A recent study in HCC revealed that LATS1 was methylated and lowly expressed in HCC, and low LATS1 expression causes YAP activation, which leads to its activation into the nucleus. Activated YAP activates MAGL and promotes the malignant development of tumors^22^. As a core factor of the Hippo signaling pathway, LATS1 plays a crucial role in regulating the expression of the downstream oncogene YAP. Recently, the absence of LATS1 has been reported to result in the formation of various types of cancers, including gliomas^23^, gastric cancer^13^, and metastatic prostate cancer^24^. Moreover, LATS1 activity is highly suppressed by CpG island methylation in renal cell carcinoma and causes low LATS1 expression in 46.7% (14/30) of renal carcinoma tissues, indicating that LATS1 hypermethylation plays an important role in the downregulation of LATS1 in renal carcinoma^8^. This finding is consistent with the results of the present study in HCC. Importantly, the present study demonstrated that LATS1 is significantly downregulated in HCC and that low LATS1 expression is closely related to poor patient prognosis. Furthermore, low LATS1 expression was inversely correlated with OS. These data suggested that LATS1 is important for tumor deterioration and plays a crucial role in tumor proliferation and recurrence.

DNA methylation is catalyzed by DNA methyltransferases using *S*-adenosylmethionine as a methyl donor^25^. DNMT3B, a de novo methyltransferase, can completely methylate the promoter region of genes and it is highly expressed and frequently upregulated in many malignancies, such as leukemia^26^, melanoma^27^, and bladder cancer^28^. Additionally, DNMT3B induces MEG3 promoter hypermethylation and results in its decreased expression, which reduces its binding to c-Jun and increases c-Jun inhibition of PHLPP1 transcription. These effects lead to Akt/p70S6K/S6 axis activation, and human bronchial malignant transformation of epithelial cells^29^. Demethylation drugs that inhibit DNMT3B expression reduce the degree of methylation of tumor suppressor genes, thus restoring the expression of these genes and thereby significantly decreasing the proliferation and invasion ability of tumor cells^14^. Currently, demethylation drugs acting on DNMT3B, such as decitabine, have been used safely in humans alone or in combination with chemotherapy^30^. In the present work, we demonstrated that miR-29c-3p repressed DNMT3B expression in HCC, leading to increased LATS1 expression. Our study demonstrated high DNMT3B expression in HCC which is closely related to poor patient prognosis.

With deepening understanding of miRNAs, scientists have found that miRNAs are widely involved in the regulation of genes that cause normal cell malignant transformation and promote tumor proliferation, metastasis, and recurrence^31^. An important mechanism underlying this is their contribution to aberrant DNA hypermethylation through regulating DNMTs. Increased attention has been focused on the ability of miR-29c-3p to participate in gene expression through the regulation of DNMT3B^31,32^. MiR-29c-3p binds to specific sites of the DNMT3B 3′-non-coding mRNA region in an incomplete complementary manner, which mediates degradation of DNMT3B mRNA and inhibits protein translation^33,34^. Abnormal expression of miR-29c-3p is closely related to the development of tumors^35^. Concerning the tumor suppressive role of miR-29c-3p in cancer, it has been demonstrated to revert aberrant methylation by targeting DNMT3B and inhibits metastasis by targeting integrin beta1 and matrix metalloproteinase 2 in lung cancer^36^. In our experimental setting, we confirmed that miR-29c-3p mediates LATS1 gene methylation via DNMT3B, which contributes to the malignant development of HCC. Restoration of DNMT3B expression blocked the miR-29c-3p-mediated inhibitory effects on HCC, which inactivates the Hippo signaling pathway. These data indicated that miR-29c-3p plays an important role in the malignant progression of HCC via DNMT3B-mediated hypermethylation and downregulation of LATS1. In addition, interestingly, miR-29 can regulate the expression of PTEN as a downstream target gene of YAP^37^. These findings, combined with our existing results, suggest that miR-29 might function both upstream and downstream of the Hippo pathway. We are curious as to whether a regulatory mechanism exists in different tumor microenvironments that causes miR-29 to play a different role in tumor and the Hippo signaling pathway. This fascinating and puzzling aspect will be the next step for our team to explore and research.

In summary, our findings demonstrated that miR-29c-3p acts as a tumor suppressive miRNA in HCC. In addition, miR-29c-3p inhibits the expression of DNMT3B and promotes LATS1 demethylation, which restores its expression and subsequently activates the Hippo signaling pathway to inhibit the malignant development of HCC. Our findings provide new insight into the molecular pathogenesis of HCC and identify miR-29c-3p and its target genes as novel therapeutic candidate targets for HCC.

## Supplementary information

Supplementary Figure 1

Supplementary Table 1

Supplementary Table 2

Supplementary Table 3

Supplementary Table 4

Supplementary Table 5
