# Supplementary figures and images for: miR-29c-3p regulates DNMT3B and LATS1 methylation to inhibit tumor progression in hepatocellular carcinoma
Source: Cell Death Dis. 2019 Jan 18;10(2):48. doi: 10.1038/s41419-018-1281-7 (PMC6362005; doi:10.1038/s41419-018-1281-7)

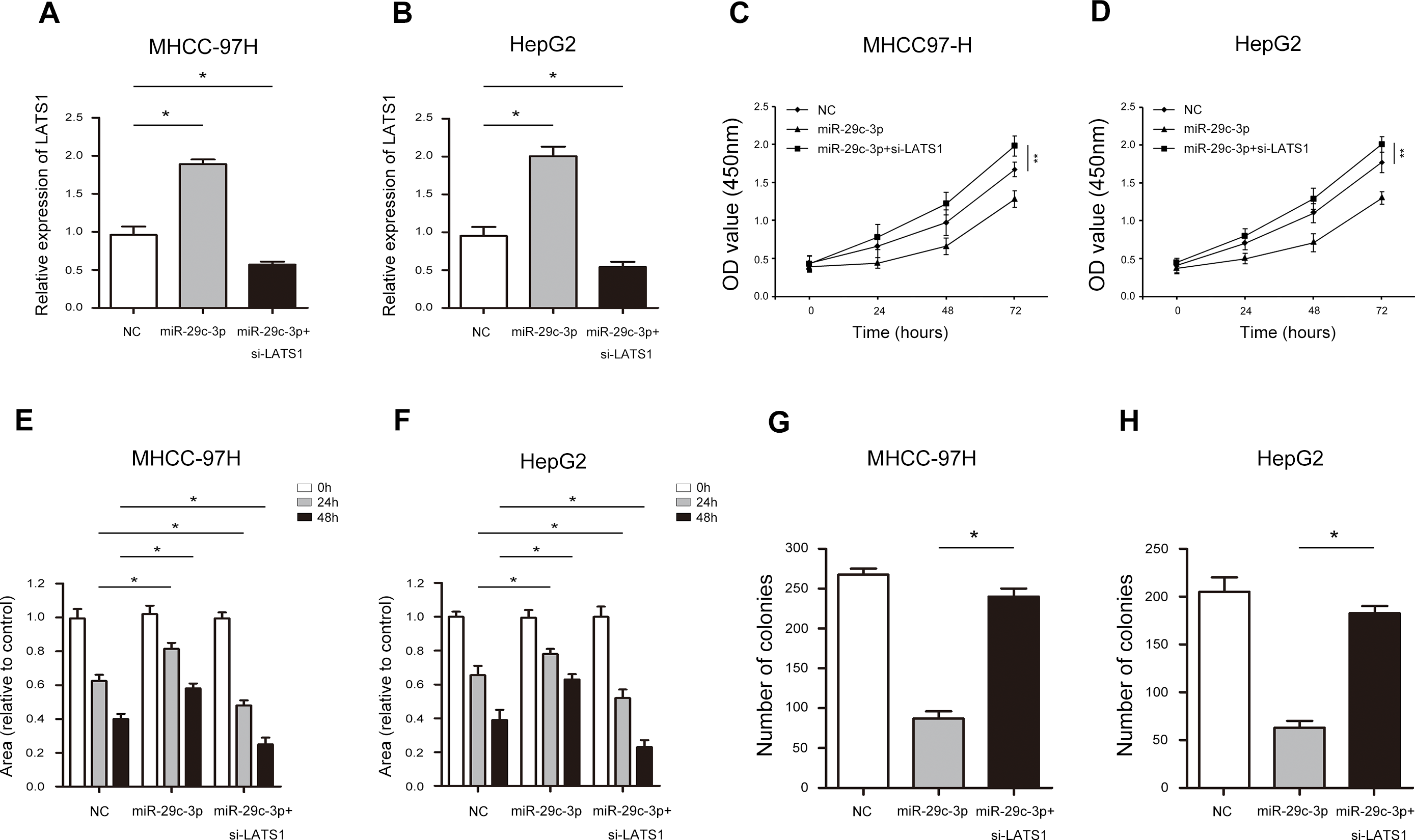

Supplement: Supplementary file 1 — Supplementary Figure 1 [file 41419_2018_1281_MOESM1_ESM.tif]
