# Supplementary material for: miR-29c-3p regulates DNMT3B and LATS1 methylation to inhibit tumor progression in hepatocellular carcinoma
Source: Cell Death Dis. 2019 Jan 18;10(2):48. doi: 10.1038/s41419-018-1281-7 (PMC6362005; doi:10.1038/s41419-018-1281-7)
Supplement: Supplementary file 2 — Supplementary Table 1 [file 41419_2018_1281_MOESM2_ESM.docx]

**Supplementary Table 1 Description of primers used for qRT-PCR**

**Geng name**  **Sequences (forward and reverse)**

miR-29c-3p F 5’-ACACTCCAGCTGGTAGCACCATTGAAAT-3’

R 5’-TGGTGTCGTGGAGTCG-3’

LATS1 F 5’- GTTAAGGGGAGAGCCAGGTCCTT-3’

R 5’-TCAAGGAAGTCCCCAGGACTGT-3’

DNMT3B F 5’-GAGTCCATTGCTGTTGAACCG-3’

R 5’-ATG TCCCTCTTGTCGCCAACCT-3’

GAPDH F 5’-GGAGCGAGATCCCTCCAAAAT-3’ R 5’-GGCTGTTGTCATACTTCTCATGG-3’
