# Supplementary material for: miR-29c-3p regulates DNMT3B and LATS1 methylation to inhibit tumor progression in hepatocellular carcinoma
Source: Cell Death Dis. 2019 Jan 18;10(2):48. doi: 10.1038/s41419-018-1281-7 (PMC6362005; doi:10.1038/s41419-018-1281-7)
Supplement: Supplementary file 3 — Supplementary Table 2 [file 41419_2018_1281_MOESM3_ESM.docx]

**Supplementary Table 2 Description of primers used for methylation specific PCR**

**Geng name**  **Sequences (forward and reverse)**

LATS1 (M primers) F 5’-TCGTTTTGTCGTTTAGGTTGG-3’

R 5’-CGACGTAATAACGAACGC-3’

LATS1 (U primers) F 5’-TAGGTTGGAGTGTGGTGGT-3’

R 5’-CCCAACATAATAACAAACACCT-3’
