# Supplementary material for: miR-29c-3p regulates DNMT3B and LATS1 methylation to inhibit tumor progression in hepatocellular carcinoma
Source: Cell Death Dis. 2019 Jan 18;10(2):48. doi: 10.1038/s41419-018-1281-7 (PMC6362005; doi:10.1038/s41419-018-1281-7)
Supplement: Supplementary file 4 — Supplementary Table 3 [file 41419_2018_1281_MOESM4_ESM.docx]

**Table 3 The correlations of DNMT3B and LATS1 with clinicopathological features of HCC patients**

| Variables *n* HCC *P* *n* HCC *P*  DNMT3B DNMT3B LATS1 LATS1  low high low high |
| --- |

150 (n＝43) （n＝107） 150 (n＝97) （n＝53）

| Age (yr)  ＜50 81 23 58 0.936 62 39 23 0.705  ≥50 69 20 49 88 58 30  Sex  Female 33 10 23 0.814 53 36 17 0.537  Male 117 33 84 97 61 36  Tumor size（cm）  ≤5 60 31 29 **＜0.001** 86 47 39 **0.003**  ＞5 90 12 78 64 50 14  AFP (ng/ml)  ≤20 64 17 47 0.623 53 34 19 0.922  ＞20 86 26 60 97 63 34  Liver cirrhosis  Presence 93 27 66 0.899 63 43 20 0.434  Absence 57 16 41 87 54 33  HBsAg  Positive 120 36 84 0.470 107 72 35 0.289  Negative 30 7 23 43 25 18  TNM stage  I/II 50 17 33 0.307 58 25 33 **＜0.001**  III/IV 100 26 74 92 72 20  Vascular invasion  Presence 84 15 69 **＜0.001** 61 39 22 0.877  Absence 66 28 38 89 58 31  Multiplicity  Single 91 27 64 0.736 73 43 30 0.151  Multiple (≥2) 59 16 43 77 54 23  Intrahepatic  metastasis  Presence 78 13 65 **0.001** 84 69 15 **＜0.001**  Absence 72 30 42 66 28 38 |
| --- |
