# Supplementary material for: miR-29c-3p regulates DNMT3B and LATS1 methylation to inhibit tumor progression in hepatocellular carcinoma
Source: Cell Death Dis. 2019 Jan 18;10(2):48. doi: 10.1038/s41419-018-1281-7 (PMC6362005; doi:10.1038/s41419-018-1281-7)
Supplement: Supplementary file 5 — Supplementary Table 4 [file 41419_2018_1281_MOESM5_ESM.docx]

**Table 4 Univariate and multivariate analysis of different prognostic variables of**

**DNMT3B with OS**

*n* Univariate analysis Multivariate analysis model

Variables HR 95% CI  *P* HR 95% CI  *P*

Sex 1.021 0.563-1.965 0.594

Female 33

Male 117

Age (yr) 1.624 0.364-1.301 0.606

＜50 81

≥50 69

AFP(ng/ml) 1.065 0.338-1.457 0.631

≤20 64

＞20 86

HBsAg 2.364 0.738-2.902 0.439

Positive 120

Negative 30

Liver Cirrhosis 0.961 0.536-1.934 0.506

Presence 93

Absence 57

TNM stage 0.763 0.791-2.014 0.863

I/II 50

III/IV 100

Tumor size (cm) 1.294 1.031-2.496 **0.037** 0.968 1.308-3.467 **＜0.001**

≤5 60

＞5 90

Multiplicity 0.769 0.589-2.108 0.676

Single 91

Multiple (≥2) 59

Intrahepatic 0.893 0.893-1.846 0.693

Metastasis

Presence 78

Absence 72

Vascular Invasion 1.483 1.673-3.746 **0.021** 2.369 1.361-4.697  **0.016**

Presence 84

Absence 66

DNMT3B expression 2.126 1.396-2.943 **0.003** 2.763 1.934-5.409 **0.011**

High 107

Low 43

*HR* hazard rate, *CI* confidence interval
