# Supplementary material for: miR-29c-3p regulates DNMT3B and LATS1 methylation to inhibit tumor progression in hepatocellular carcinoma
Source: Cell Death Dis. 2019 Jan 18;10(2):48. doi: 10.1038/s41419-018-1281-7 (PMC6362005; doi:10.1038/s41419-018-1281-7)
Supplement: Supplementary file 6 — Supplementary Table 5 [file 41419_2018_1281_MOESM6_ESM.docx]

**Table 5 Univariate and multivariate analysis of different prognostic variables of**

**LATS1 with OS**

*n* Univariate analysis Multivariate analysis model

Variables HR 95% CI *P*  HR 95% CI  *P*

Sex 1.349 0.963-1.639 0.863

Female 53

Male 97

Age (yr) 1.434 0.537-1.968 0.539

＜50 62

≥50 88

AFP(ng/ml) 0.839 1.728-3.648 0.799

≤20 53

＞20 97

HBsAg 1.948 0.694-2.902 0.964

Positive 107

Negative 43

Liver Cirrhosis 1.281 0.397-1.674 0.756

Presence 63

Absence 87

TNM stage 1.580 1.013-2.769 **0.033** 1.186 1.556-3.690  **0.040**

I/II 58

III/IV 92

Tumor size (cm) 1.890 0.769-2.699 0.708

≤5 86

＞5 64

Multiplicity 0.893 1.304-3.864 0.538

Single 73

Multiple (≥2) 77

Intrahepatic Metastasis 1.253 1.309-3.343  **0.024** 1.625 1.026-2.964 **0.017**

Presence 84

Absence 66

Vascular Invasion 1.074 0.899-2.480 **0.021** 1.768 0.863-1.837 **0.037**

Presence 61

Absence 89

LATS1 expression 0.426 0.887-2.093 **0.007** 0.763 1.324-3.574 **0.015**

High 53

Low 97

*HR* hazard rate, *CI* confidence interval
